# Diversity of endophytic bacterial microbiota in grapevine shoot xylems varies depending on wine grape-growing region, cultivar, and shoot growth stage

**DOI:** 10.1038/s41598-022-20221-8

**Published:** 2022-09-21

**Authors:** Kazuhiro Hamaoka, Yoshinao Aoki, Sayuri Takahashi, Shinichi Enoki, Kosuke Yamamoto, Keisuke Tanaka, Shunji Suzuki

**Affiliations:** 1 Laboratory of Fruit Genetic Engineering, The Institute of Enology and Viticulture, University of Yamanashi, 1-13-1 Kitashin, Kofu, Yamanashi 400-0005 Japan; 2 Department of Molecular Microbiology, Faculty of Life Sciences, Tokyo University of Agriculture, 1-1-1 Sakuragaoka, Setagaya-ku, Tokyo, 156-8502 Japan; 3 NODAI Genome Research Center, Tokyo University of Agriculture, 1-1-1 Sakuragaoka, Setagaya-ku, Tokyo, 156-8502 Japan

**Keywords:** Ecology, Microbiology, Plant sciences

## Abstract

Next-generation sequencing technology may clarify microbiota that are as yet poorly understood in the soil, the rhizosphere, and the phyllosphere of vineyards. To provide new information on the interaction between grapevine and microorganisms, we focused on the endophytic microbiota in grapevine. We performed endophytic microbiome analysis of the shoot xylems of four cultivars, *Vitis vinifera* cvs. Chardonnay, Pinot Noir, Cabernet Sauvignon, and *Vitis* sp. cv. Koshu, grown in eleven vineyards in Japan. The number of endophytic fungal species was small in the grapevine shoot xylems and could not be analyzed further, whereas a total of 7,019,600 amplicon sequences (46,642–285,003 per shoot xylem) and 1305 bacterial operational taxonomic units were obtained by analysis of the V3–V4 region of the bacterial 16S rRNA gene. Gammaproteobacteria was predominant in the shoot xylems at the shoot elongation stage irrespective of the cultivar, whereas Alphaproteobacteria and Oxyphotobacteria were predominant at véraison. Actinobacteria, Bacteroidia, Bacilli, and Clostridia were also detected in the shoot xylems. The endophytic bacterial microbiota in Koshu and Pinot Noir shoot xylems were similar irrespective of the grapevine-growing region. In contrast, the endophytic bacterial microbiota in Chardonnay and Cabernet Sauvignon showed diversity and complexity among grapevine-growing regions. Alpha diversity analysis revealed that Koshu shoot xylems had a higher diversity of endophytic bacterial microbiota than Pinot Noir, Chardonnay, and Cabernet Sauvignon shoot xylems, and that grapevine shoot xylems at the shoot elongation stage had a higher diversity of endophytic bacterial microbiota than those at véraison. Principal coordinate analysis (PCoA) demonstrated that the profiles of the endophytic bacterial microbiota in grapevine shoot xylems at véraison were relatively uniform compared with those at the shoot elongation stage. Multidimensional scaling analysis showed that the plots of all cultivars were generally apart from each other at the shoot elongation stage and then became close to each other at véraison. The plots of all grapevine-growing regions cultivating Koshu were close to each other, whereas those of grapevine-growing regions cultivating Chardonnay and Cabernet Sauvignon were apart from each other. The findings of this study suggest that the endophytic bacterial microbiota in grapevine shoot xylems varied depending on the cultivar and the grapevine-growing region even for the same cultivars, and that the microbiota fluctuated depending on the shoot growth stage.

## Introduction

Environmental conditions in each grapevine-growing region may affect the quality of grape berries and cause a diversity in wine flavor and aroma even for the same grape cultivars. Soil composition^1^, topography^2^, and climate^1,3,4^ in grapevine-growing regions have been studied worldwide to understand the relationship of these factors with grape berry and wine qualities. In Japan, the characteristics of berries of Koshu, which is a Japanese indigenous *Vitis* sp., vary among the grapevine-growing regions having different climatic conditions^5^. For example, the average monthly air temperature showed a strong relationship with total soluble solid content, titratable acid content, fresh berry weight, total phenol content, caftaric acid content, and coutaric acid content in Koshu berries. Total integrated solar radiation also showed a positive correlation with soluble solid content and pH and a negative correlation with titratable acid content in Koshu berries. However, multiple complicatedly intertwined geological and meteorological factors have made it difficult to understand the effects of environmental conditions on the quality of grape berries and wine flavor and aroma.

Some scientists have been paying attention to soil microbiota in vineyards to understand the diversity of grape berry and wine qualities among vineyards. Soils contain large numbers of fungi, bacteria, and archaea. Most soil microorganisms remain unidentified and may have unknown physiological and ecological attributes in plant–soil–microorganisms interactions^6^. Interestingly, environmental factors such as soil composition^7^, topography^8^, and climate^9^ synergistically affect soil microbiota. Soil type^10^ and pH^11^ are the key factors determining bacterial microbiota in arable soils. Topography and climate, such as rainfall pattern and temperature, affect soil microbiota in vineyards through their impacts on soil^8^. Thus, distinctive soil microbiota in each vineyard may result in the diversity of grape berry and wine qualities among grapevine-growing regions. However, the complete composition of soil microbiota cannot be determined using current technologies owing to the enormous number of microbial species and populations in soils^12^. The relationship between soil microbiota and grape berry and wine qualities requires further examination.

An endophyte is an endosymbiotic bacterium^13^ or fungus^14^ within a plant. Endophytism is a mutualistic and win–win relationship between a host plant and an endophyte^15^. Endophytes frequently alter the physiological status of host plants. Some endophytes stimulate root growth by secreting plant hormones within the host plant^16^. Colonization by the endophytic fungus *Neotyphodium lolii* in perennial ryegrass affects the phenolic content in the host grass^17^. Endophytes also confer tolerance to environmental stresses in host plants. Reactive oxygen species produced by endophytic fungi protect host plants from oxidative stress^18^. Endophytic Actinobacteria isolated from healthy wheat tissues enhances the disease resistance of *Arabidopsis* plants by activating systemic defense pathways^19^. Thus, endophytic microbiota are an active component of host plants’ responses to biotic and abiotic stresses. Optimal endophytic microbiota make host plants healthy, whereas plants with imbalanced endophytic populations may be in poor health.

Recently, endophytic microbiota in grapevine trunk^20^ and cordon, shoot, and sap^21^ have been receiving much attention. Minor differences in endophytic bacterial microbiota were detected between shoots of Merlot and Chardonnay cultivated in northern Italy^22^. Because the diversity of endophytic microbiota within the grapevine may be affected by environmental factors^23^, the health of the grapevine may also be affected by the diversity and composition of endophytic microbiota associated with the grapevine^24–26^. We report here the diversity of endophytic bacterial microbiota in the shoot xylems of four cultivars (*Vitis vinifera* cvs. Chardonnay, Pinot Noir, Cabernet Sauvignon, and *Vitis* sp. cv. Koshu) grown in eleven vineyards in Japan. We demonstrate that the profiles of endophytic bacterial microbiota in grapevine shoot xylems varied depending on the cultivar and the grapevine-growing region even for the same cultivars, and that they fluctuated depending on the shoot growth stage.

## Materials and methods

### Vineyards

Plant samples were collected from eleven vineyards located in major grapevine-growing regions in Japan, namely, Urausu in Hokkaido Prefecture, Minamisanriku in Miyagi Prefecture, Katsunuma in Yamanashi Prefecture, Kofu in Yamanashi Prefecture, Kai in Yamanashi Prefecture, Komoro in Nagano Prefecture, Ueda in Nagano Prefecture, Izumo in Shimane Prefecture, Shobara in Hiroshima Prefecture, Saijo in Hiroshima Prefecture, and Omishima in Ehime Prefecture (Fig. S1). The latitude, longitude, and elevation of each vineyard are shown in Supplementary Table 1.

Weather data of each vineyard were obtained from the website of Japan Meteorological Agency (https://www.jma.go.jp/jma/menu/menureport.html). Growing degree days (GDDs, base threshold of 10 °C) and precipitation from April 1 to October 31, 2020 are summarized in Supplementary Table 2.

### Plant samples

We cannot deny the possibility that the profiles of endophytic microbiota in grapevine shoot xylems varies among shoot samples collected from the same grapevine plant. To determine whether the profiles of endophytic microbiota are equivalent between shoot samples collected from the same grapevine plant or between shoot samples collected from different grapevine plants cultivated in the same vineyard, *Vitis vinifera* cv. Chardonnay and *Vitis* sp. cv. Koshu, cultivated in the experimental vineyard of The Institute of Enology and Viticulture, University of Yamanashi, Yamanashi, Japan, were used and two grapevine plants were analyzed for each cultivar. The grapevine plants were grafted onto Kober 5BB and were approximately 30 years old. Two shoot samples were randomly collected from each grapevine on December 11, 2019.

We gained permission to collect and use grapevine shoots from wineries and research institution as shown in the acknowledgements. We collected one shoot sample from a grapevine plant, at two different shoot growth stages of each cultivar grown in the eleven vineyards located in major grapevine-growing regions in Japan. Briefly, at the shoot elongation stage (the middle of May to the beginning of June) and véraison (the end of July to the end of August) in 2020, samples of shoots of *V. vinifera* cvs. Chardonnay, Pinot Noir, Cabernet Sauvignon, and *Vitis* sp. cv. Koshu were collected from the vineyards. A total of 52 shoot samples were obtained for endophytic microbiota analysis. The collection date and the cultivars for each vineyard are summarized in Supplementary Table 1.

### Extraction of endophytic microbial DNA from the shoot xylem of the first internode

The surface of the first internode from the bottom of a shoot was sterilized in 0.1% (v/v) sodium hypochlorite for 3 min at room temperature. The bark and the epidermal tissue were peeled off from the shoot using a sterilized knife. Xylem shavings were obtained using a sterilized grater. One gram of xylem shavings was added to a sterilized 100 mL flask containing 40 mL of phosphate buffer solution (pH 7.4). After shaking at 130 rpm for 3 h at 25 °C, the solution was filtered through a sterilized cotton gauze. The filtrate was transferred to a 50 mL centrifuge tube and centrifuged at 3,000 rpm for 10 min at room temperature. The supernatant was collected into a new centrifuge tube and centrifuged at 10,000 rpm for 10 min at room temperature. DNA was extracted from the pellet using DNeasy PowerSoil Kit (Qiagen, Hilden, Germany) and used as the endophytic microbial DNA.

### Amplicon library of bacterial 16S rRNA gene

rRNA metagenomic analysis of the endophytic microbial DNA was performed by NODAI Genome Research Center (Tokyo, Japan). In a preliminary experiment, the rRNA-ITS region of fungal rRNA was rarely detected in the endophytic microbial DNA. Thus, in this study, bacterial 16S rRNA metagenomic analysis was performed using the endophytic microbial DNA. Briefly, the amplicon library in the V3-V4 hypervariable region of the bacterial 16S rRNA gene was constructed in accordance with the 16S Metagenomic Sequencing Library Preparation protocol (Illumina, San Diego, CA). First-round PCR was performed using 2X KAPA HiFi HotStart ReadyMix (Kapa Biosystems, Woburn, MA) and Illumina overhang adapters and universal primers. The nucleotide sequences of the adapters and the primers used in this study were as follows: forward primer, 5′-TCGTCGGCAGCGTCAGATGTATAAGAGACAGCCTACGGGNGGCWGCAG-3′ (underline, adapter sequence); reverse primer, 5′-GTCTCGTGGGCTCGGAGATGTGTATAAGAGACAGGACTACHVGGGTATCTAATCC-3′ (underline, adapter sequence). First-round PCR conditions were as follows: after incubation at 95 °C for 3 min, PCR amplification was performed for 25 cycles at 95 °C for 30 s, 55 °C for 30 s, and 72 °C for 30 s with a final extension step at 72 °C for 5 min. After cleaning the first-round PCR product using an AMPure XP kit (Beckman Coulter, Brea, CA), the second-round PCR was performed using 2X KAPA HiFi HotStart ReadyMix (Kapa Biosystems) and Nextera XT Index Kit (Illumina). Second-round PCR conditions were as follows: after incubation at 95 °C for 3 min, PCR amplification was performed for 8 cycles at 95 °C for 30 s, 55 °C for 30 s, and 72 °C for 30 s with a final extension step at 72 °C for 5 min. After cleaning the second-round PCR product using the AMPure XP kit, the quality and quantity of the amplicon libraries were evaluated using Agilent 2200 TapeStation (Agilent Technologies, Santa Clara, CA), and KAPA Library Quantification Kit (KAPA Biosystems) and Step-One-Plus real-time PCR system (Applied Biosystems, Foster City, CA), respectively.

### Bacterial 16S rRNA metagenomic analysis

The amplicon libraries were sequenced by 2 × 300 bp paired-end reading using the MiSeq platform (Illumina). The sequenced read data were submitted to the DDBJ Read Archive (https://www.ddbj.nig.ac.jp/dra/index.html; accession number DRA012630, hold date: August 24, 2023).

Data analysis and statistical analysis were performed using QIIME2 pipeline version 2019.10^27^. Primer trimming, quality check, and chimera filtering were performed using the q2-dada2 plugin as follows^28^: –p-trim-left-f = 17, –p-trim-left-r = 21, –p-trunc-len-f = 0, and –p-trunc-len-r = 290. The sequenced reads were rarefied at a depth of 46,642 using repeated rarefaction with the q2-diversity plugin. Taxonomic annotation was performed at 99% similarity using the classify-sklearn naïve Bayes taxonomy classifier via the q2-feature-classifier plugin in QIIME2 pipeline version 2019.10 with the SILVA 132 database as reference^29^. Alpha diversity analysis was performed by determining operational taxonomic units (OTUs), Chao1 index, and Shannon index in accordance with a method published previously^28^. To further analyze the diversity of endophytic bacterial microbiota in shoot samples, beta diversity analysis with principal coordinate analysis (PCoA) and weighted UniFrac distance, permutational multivariate analysis of variance (PERMANOVA), and cluster analysis with multidimensional scaling (MDS) were also performed in accordance with methods published previously^30–32^.

### Ethics statement

The collection of the plant material and related experiments complies with relevant institutional, national, and international guidelines. All methods were carried out in accordance with relevant guidelines and regulations.

## Results

### Preliminary experiment using grapevine shoot samples

To determine whether the profiles of endophytic bacterial microbiota vary widely between shoot samples collected from the same grapevine plant or between shoot samples collected from different grapevine plants of the same cultivar grown in the same vineyard, a preliminary experiment was performed. Microbiome analysis demonstrated that the profiles of endophytic bacterial microbiota were similar between two shoot samples collected from the same Chardonnay or Koshu grapevine plant (Fig. S2). In addition, the profiles of endophytic bacterial microbiota in shoot samples collected from different Chardonnay or Koshu grapevine plants cultivated in the same vineyard were also similar (Fig. S2). These results suggest that the profiles of endophytic bacterial microbiota in shoot samples collected from different grapevine plants of the same cultivar grown in the same vineyard were uniform. On the basis of this finding, we collected one shoot sample from a grapevine plant, at two different shoot growth stages (shoot elongation stage and véraison), of each cultivar grown in the eleven vineyards located in major grapevine-growing regions in Japan.

### Weather data

GDDs from April 1 to October 31, 2020 demonstrated that Minamisanriku and Ueda belonged to Region III on the Winkler Index and that Komoro, Shobara, and Saijo belonged to Region IV on the Winkler Index (Supplementary Table 2). Only Urausu belonged to Region II on the Winkler Index. Five vineyards including Kofu, Kai, Katsunuma, Izumo, and Omishima belonged to Region V on the Winkler Index, suggesting that Chardonnay, Pinot Noir, and Cabernet Sauvignon were cultivated under extremely high temperatures in those vineyards. Precipitation from April 1 to October 31, 2020 exceeded 1700 mm in Shobara, the highest among the vineyards (Supplementary Table 2).

### Amplicon sequences collected from grapevine shoot xylems

**Figure 1:**
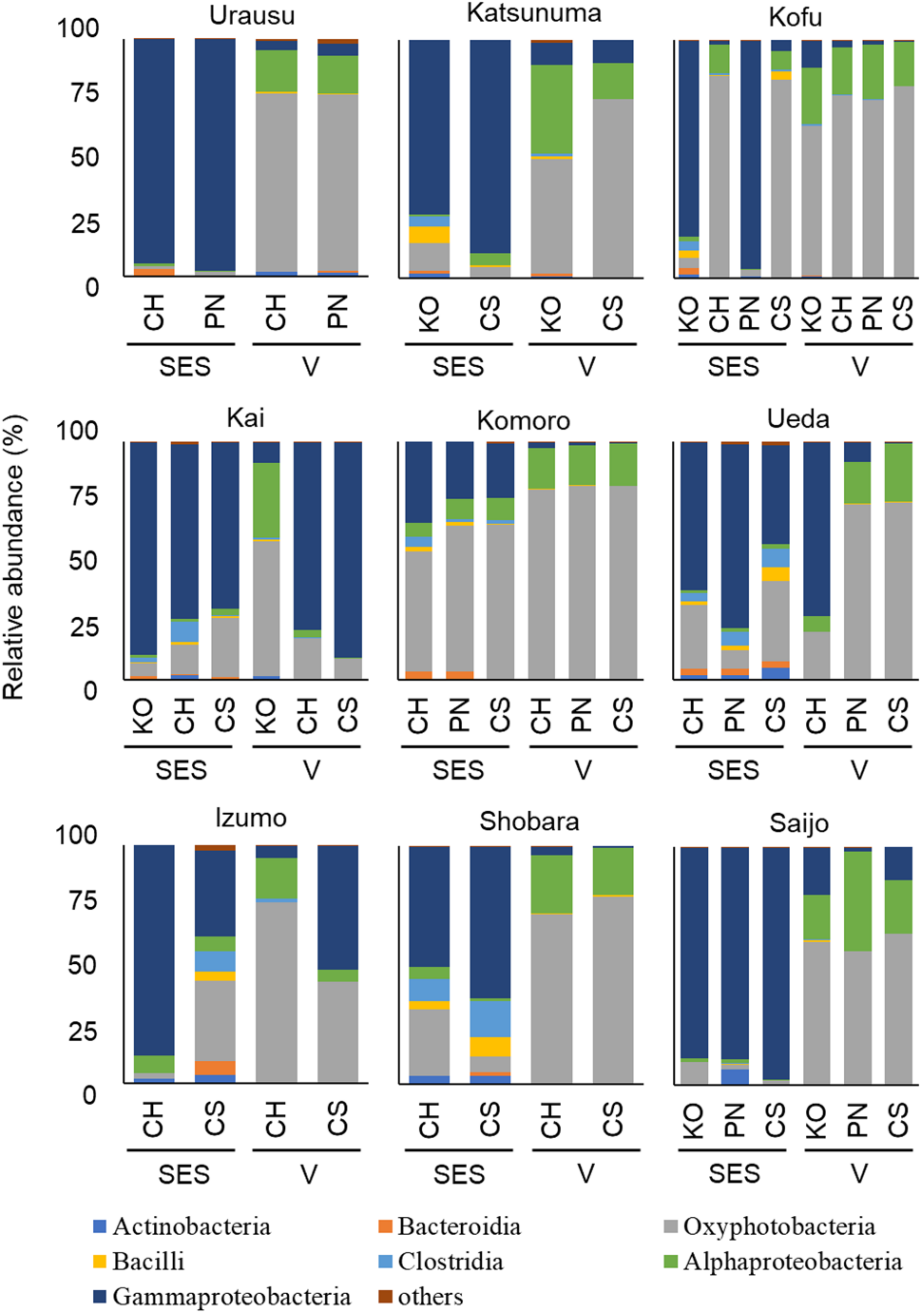
Endophytic bacterial microbiota in shoot xylems of cultivars grown in the same vineyard. Endophytic bacterial microbiota in the shoot xylems of each cultivar collected from nine vineyards were identified and evaluated at the class level. Data are presented as relative abundance (%). *KO* Koshu, *CH* Chardonnay, *CS* Cabernet Sauvignon, *PN* Pinot Noir, *SES* shoot elongation stage, *V* véraison.

A total of 7,019,600 amplicon sequences from 52 samples were collected (Supplementary Table 3). We identified a total of 1305 OTUs on the basis of the conventional criterion of 99% sequence similarity. Irrespective of cultivar, grapevine-growing region, and shoot growth stage, Alphaproteobacteria, Gammaproteobacteria*,* and Oxyphotobacteria were predominant in shoot xylems (Fig. 1). Actinobacteria, Bacteroidia, Bacilli, and Clostridia were the endophytic bacteria detected in the shoot xylems.

### Comparison of endophytic bacterial microbiota in grapevine shoot xylems of cultivars grown in the same vineyard

Shoot samples of two or more cultivars were collected from nine vineyards (Urausu, Katsunuma, Kofu, Kai, Komoro, Ueda, Izumo, Shobara, and Saijo) and evaluated (Fig. 1). Below are the detailed results for each vineyard.

### Urausu (Hokkaido Prefecture)

At the shoot elongation stage, more than 90% of endophytic bacteria in Chardonnay and Pinot Noir shoot xylems belonged to class Gammaproteobacteria. Oxyphotobacteria was also detected in the shoot xylems albeit at a very low proportion (1% and 2% in Chardonnay and Pinot Noir, respectively). At véraison, the proportion of Oxyphotobacteria increased and reached 75% and 74% in Chardonnay and Pinot Noir shoot xylems, respectively. Overall, the profiles of endophytic bacterial microbiota were very similar between Chardonnay and Pinot Noir cultivated in Urausu at each shoot growth stage.

### Katsunuma (Yamanashi Prefecture)

At the shoot elongation stage, Gammaproteobacteria was predominant in Koshu and Cabernet Sauvignon shoot xylems, although Oxyphotobacteria and Bacilli were detected as well. At véraison, the proportion of Oxyphotobacteria increased and reached 48% and 75% in Koshu and Cabernet Sauvignon shoot xylems, respectively. The proportion of Alphaproteobacteria also increased at véraison (37% and 15% in Koshu and Cabernet Sauvignon, respectively). Overall, the profiles of endophytic bacterial microbiota were similar between Koshu and Cabernet Sauvignon cultivated in Katsunuma at each shoot growth stage.

### Kofu (Yamanashi Prefecture)

Shoot samples of Koshu, Chardonnay, Pinot Noir, and Cabernet Sauvignon were collected from Kofu. At the shoot elongation stage, Gammaproteobacteria was predominant (approximately 90%) in Koshu and Pinot Noir shoot xylems, whereas more than 80% of endophytic bacteria in Chardonnay and Cabernet Sauvignon shoot xylems belonged to class Oxyphotobacteria. At véraison, the profiles of endophytic bacterial microbiota were similar among the four cultivars grown in Kofu, and Oxyphotobacteria was predominant.

### Kai (Yamanashi Prefecture)

Irrespective of the shoot growth stage, Gammaproteobacteria was predominant in Chardonnay and Cabernet Sauvignon shoot xylems. Although Gammaproteobacteria was also predominant in the Koshu shoot xylems at the shoot elongation stage, the proportions of Oxyphotobacteria and Alphaproteobacteria increased in Koshu shoot xylems at véraison (57% and 32%, respectively).

### Komoro (Nagano Prefecture)

Irrespective of the cultivar (Chardonnay, Pinot Noir, and Cabernet Sauvignon), the profiles of endophytic bacterial microbiota in shoot xylems were very similar at each shoot growth stage, and Oxyphotobacteria was predominant. More than 80% of endophytic bacteria in the shoot xylems at véraison belonged to class Oxyphotobacteria.

### Ueda (Nagano Prefecture)

The profiles of endophytic bacterial microbiota in shoot xylems at the shoot elongation stage were similar among Chardonnay, Pinot Noir, and Cabernet Sauvignon, whereas the profile in Chardonnay shoot xylems at véraison was different from those in Pinot Noir and Cabernet Sauvignon shoot xylems. Gammaproteobacteria (76%) was predominant in Chardonnay shoot xylem at véraison. In Pinot Noir and Cabernet Sauvignon shoot xylems at véraison, more than 70% of endophytic bacteria belonged to class Oxyphotobacteria.

### Izumo (Shimane Prefecture)

Unlike other vineyards, there was no similarity of profiles between cultivars (Chardonnay and Cabernet Sauvignon) and between shoot growth stages. Gammaproteobacteria and Oxyphotobacteria were predominant in Chardonnay shoot xylems at the shoot elongation stage and véraison, respectively. In Cabernet Sauvignon shoot xylems, Gammaproteobacteria (36% and 52% at the shoot elongation stage and véraison, respectively) and Oxyphotobacteria (34% and 43% at the shoot elongation stage and véraison, respectively) were predominant irrespective of the shoot growth stage.

### Shobara (Hiroshima Prefecture)

Similarly to Urausu and Katsunuma, Gammaproteobacteria was predominant in Chardonnay and Cabernet Sauvignon shoot xylems at the shoot elongation stage. The proportion of Oxyphotobacteria increased at véraison; more than 70% of endophytic bacteria in Chardonnay and Cabernet Sauvignon shoot xylems at véraison belonged to class Oxyphotobacteria. Overall, the profiles of endophytic bacterial microbiota were similar between Chardonnay and Cabernet Sauvignon cultivated in Shobara at each shoot growth stage.

### Saijo (Hiroshima Prefecture)

Similarly to Urausu, Katsunuma, and Shobara, Gammaproteobacteria (89%, 89%, and 98% in Koshu, Pinot Noir, and Cabernet Sauvignon shoot xylems, respectively) was predominant at the shoot elongation stage and Oxyphotobacteria (60%, 56%, and 63% in Koshu, Pinot Noir, and Cabernet Sauvignon shoot xylems, respectively), at véraison. Overall, the profiles of endophytic bacterial microbiota were similar among Koshu, Pinot Noir, and Cabernet Sauvignon cultivated in Saijo at each shoot growth stage.

### Comparison of endophytic bacterial microbiota in grapevine shoot xylems of cultivars grown in different vineyards

**Figure 2:**
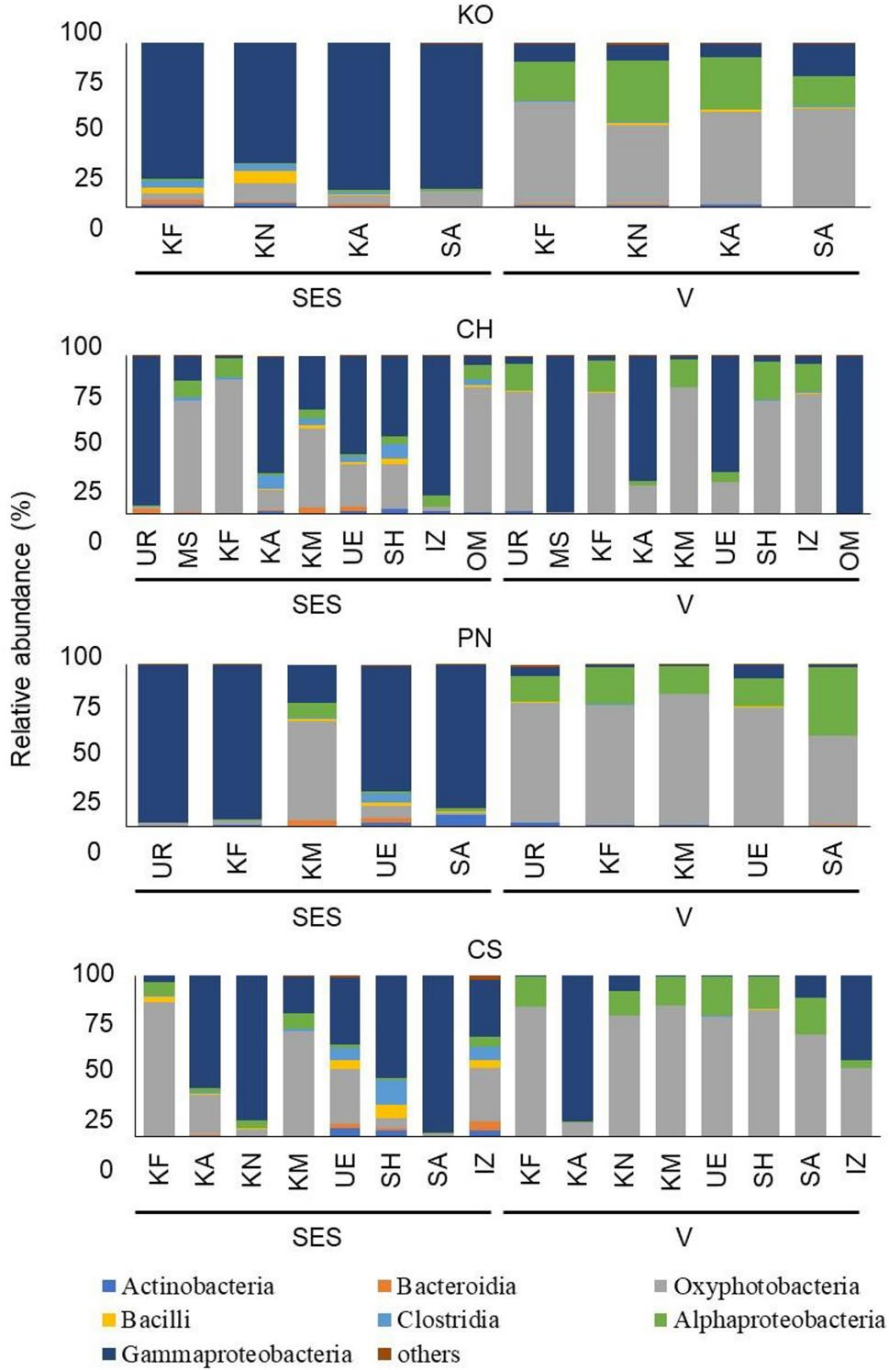
Endophytic bacterial microbiota in shoot xylems of cultivars grown in the different vineyards. Endophytic bacterial microbiota in the shoot xylems of each cultivar collected from different vineyards were identified and evaluated at the class level. Data are presented as relative abundance (%). *KO* Koshu, *CH* Chardonnay, *CS* Cabernet Sauvignon, *PN* Pinot Noir, *SES* shoot elongation stage, *V* véraison, *UR* Urausu, *MS* Minamisanriku, *KF* Kofu, *KA* Kai, *KN* Katsunuma, *KM* Komoro, *UE* Ueda, *SH* Shobara, *IZ* Izumo, *SA* Saijo, *OM* Omishima.

The profiles of endophytic bacterial microbiota in the shoot xylems of Koshu, Chardonnay, Pinot Noir, and Cabernet Sauvignon cultivated in different vineyards were evaluated (Fig. 2). In Koshu shoot xylems, the profiles of endophytic bacterial microbiota were similar at each shoot growth stage irrespective of the vineyard. Gammaproteobacteria (73–89%) was predominant in Koshu shoot xylems at the shoot elongation stage, whereas Oxyphotobacteria (48–63%) and Alphaproteobacteria (19–37%) were predominant at véraison. At the shoot elongation stage, Pinot Noir cultivated in Komoro showed different diversity of endophytic bacterial microbiota from Pinot Noir cultivated in the other vineyards. At véraison, the profiles of endophytic bacterial microbiota in Pinot Noir shoot xylems were similar irrespective of the vineyard. Gammaproteobacteria (76–98%) was predominant in Pinot Noir shoot xylems at the shoot elongation stage, whereas Oxyphotobacteria (56–81%) was predominant at véraison. In contrast to Koshu and Pinot Noir, the profiles of endophytic bacterial microbiota in Chardonnay and Cabernet Sauvignon shoot xylems showed diversity and complexity among vineyards. At the shoot elongation stage, Oxyphotobacteria was predominant in Chardonnay shoot xylems at Minamisanriku (70%) and Kofu (85%), whereas Gammaproteobacteria was predominant in the other vineyards. At véraison, more than 95% of endophytic bacteria in shoot xylems of Chardonnay cultivated in Minamisanriku and Omishima belonged to class Gammaproteobacteria. In the case of Cabernet Sauvignon, although Oxyphotobacteria and Gammaproteobacteria were predominant in shoot xylems at both shoot elongation stage and véraison, their proportions drastically varied among vineyards.

### Comparison of endophytic bacterial microbiota in grapevine shoot xylems between shoot elongation stage and véraison

**Figure 3:**
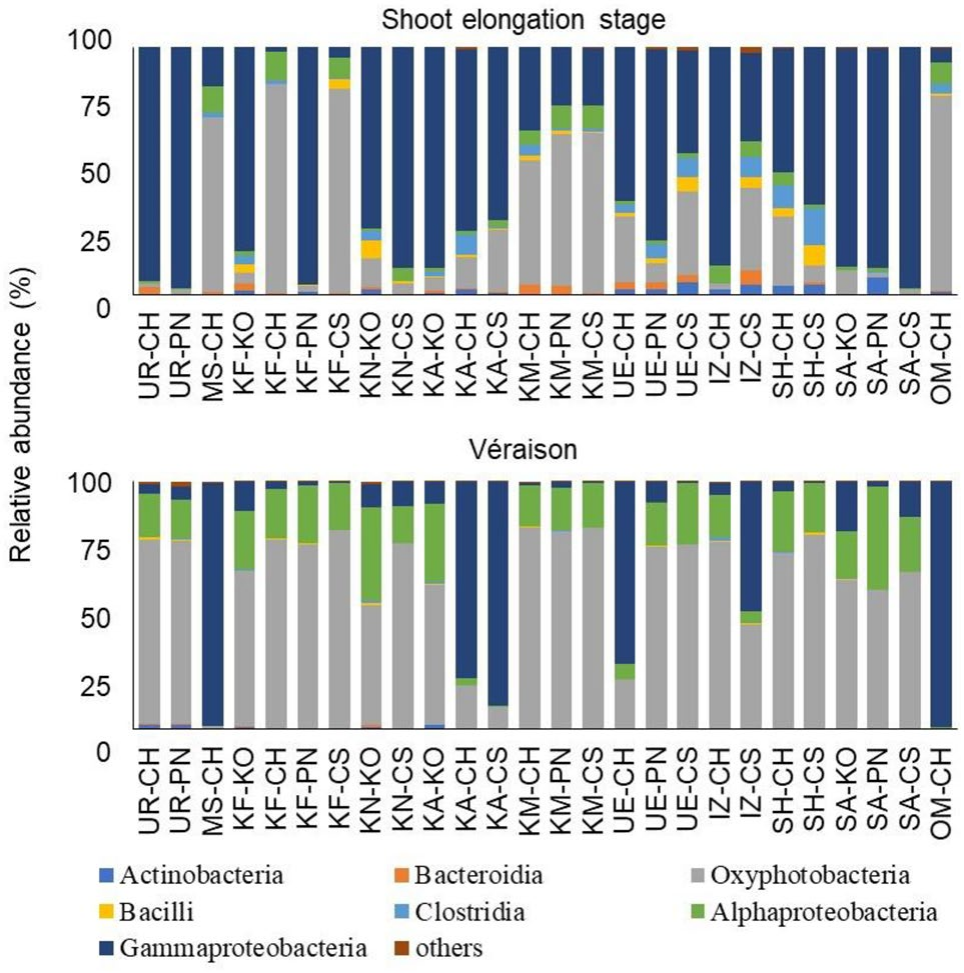
Endophytic bacterial microbiota in grapevine shoot xylems at shoot elongation stage and véraison. Endophytic bacterial microbiota in the shoot xylems collected at the shoot elongation stage and véraison were identified and evaluated at the class level. Data are presented as relative abundance (%). *UR* Urausu, *MS* Minamisanriku, *KF* Kofu, *KA* Kai, *KN* Katsunuma, *KM* Komoro, *UE* Ueda, *SH* Shobara, *IZ* Izumo, *SA* Saijo, *OM* Omishima, *KO* Koshu, *CH* Chardonnay, *CS* Cabernet Sauvignon, *PN* Pinot Noir.

The profiles of endophytic bacterial microbiota in the shoot xylems, regardless of the cultivar, at each shoot growth stage were evaluated (Fig. 3). The profiles of endophytic bacterial microbiota in grapevine shoot xylems at the shoot elongation stage were diverse and complex. Although Oxyphotobacteria and Gammaproteobacteria were predominant in the shoot xylems at the shoot elongation stage, various endophytic bacteria including those belonging to classes Actinobacteria, Bacteroidia, Bacilli, Clostridia, and Alphaproteobacteria existed in the shoot xylems as well. In contrast, the profiles of endophytic bacterial microbiota in grapevine shoot xylems at véraison showed far less variation than those at the shoot elongation stage. Oxyphotobacteria, Alphaproteobacteria, and Gammaproteobacteria accounted for more than 95% of endophytic bacteria in the shoot xylems at véraison.

### Alpha diversity of endophytic bacterial microbiota in grapevine shoot xylems

**Figure 4:**
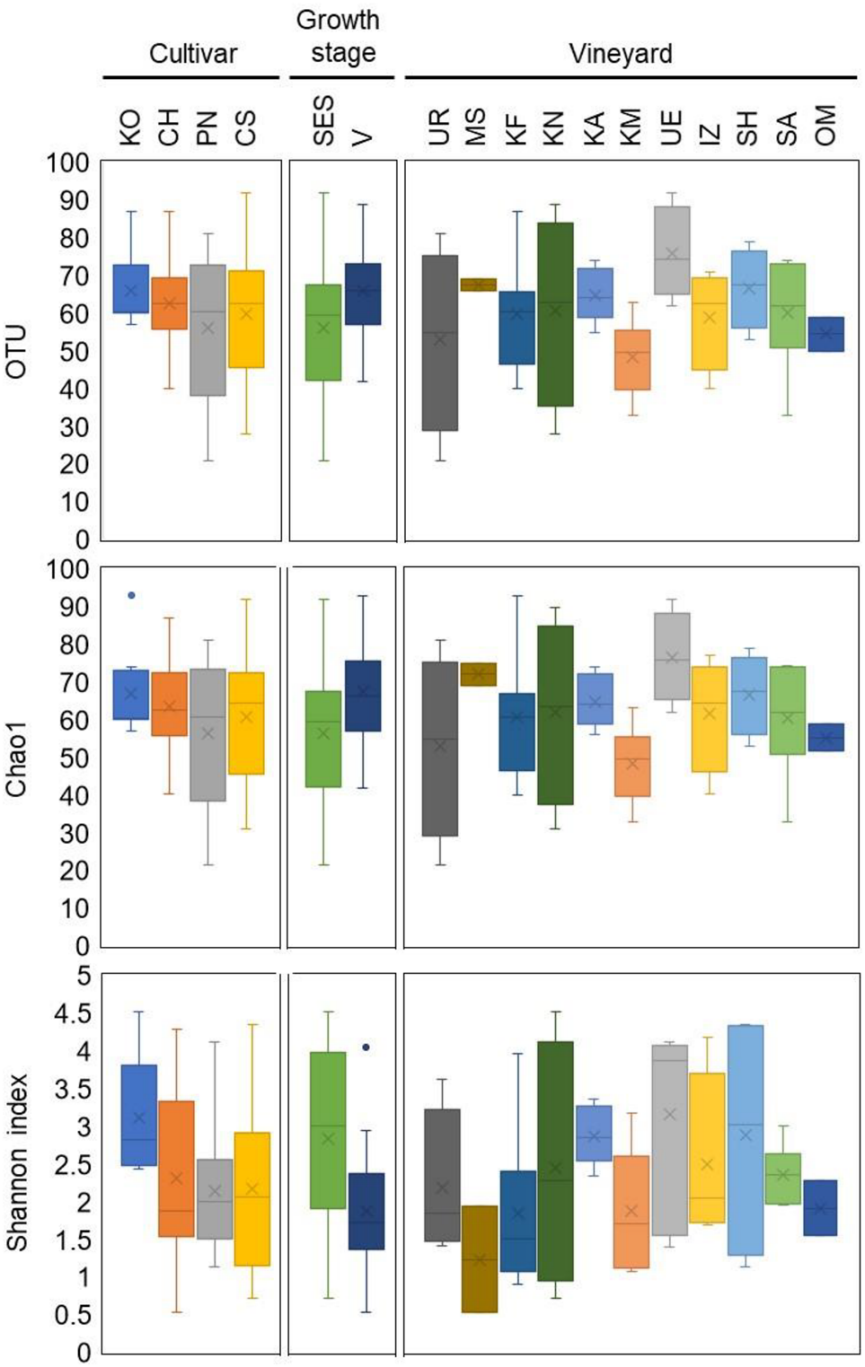
Alpha diversity of endophytic bacterial microbiota in grapevine shoot xylems. Alpha diversity analyses of cultivars, shoot growth stages, and vineyards were performed. Upper panels, OTUs; middle panels, Chao1 index; lower panels, Shannon index. Cross (×) indicates the average for each sample. *KO* Koshu, *CH* Chardonnay, *CS* Cabernet Sauvignon, *PN* Pinot Noir, *SES* shoot elongation stage, *V* véraison, *UR* Urausu, *MS* Minamisanriku, *KF* Kofu, *KA* Kai, *KN* Katsunuma, *KM* Komoro, *UE* Ueda, *SH* Shobara, *IZ* Izumo, *SA* Saijo, *OM* Omishima.

OTUs, Chao1 index, and Shannon index were used as indexes of alpha diversity of endophytic bacterial microbiota among cultivars, shoot growth stages, and vineyards (Fig. 4). The medians of OTUs were similar among the four cultivars (60.5 for Koshu and Pinot Noir, and 62.5 for Chardonnay and Cabernet Sauvignon). The medians of the Chao1 index were also comparable among the four cultivars (60 for Koshu and Pinot Noir, 63 for Chardonnay, and 65 for Cabernet Sauvignon). The median of the Shannon index (2.8) was highest for Koshu, whereas those for Pinot Noir, Chardonnay, and Cabernet Sauvignon were similar (2.0, 1.9, and 2.1, respectively). These results suggest that Koshu shoot xylems had a higher diversity of endophytic bacterial microbiota than Pinot Noir, Chardonnay, and Cabernet Sauvignon shoot xylems.

The medians of OTUs and Chao1 index at the shoot elongation stage were comparable to those at véraison. The median of the Shannon index at the shoot elongation stage (3.0) was higher than that at véraison (1.7), indicating that grapevine shoot xylems at the shoot elongation stage had a higher diversity of endophytic bacterial microbiota than those at véraison.

The medians of OTUs and Chao1 index were the highest for Ueda (74.5 and 75, respectively), whereas those were the lowest for Komoro (49.5 and 50, respectively). The medians of the Shannon index were lowest and highest for Minamisanriku (1.2) and Ueda (3.9), respectively. These results suggest that a large number of endophytic bacterial species existed in the shoot xylems of grapevine cultivated in Ueda, and that Ueda had the highest diversity of endophytic bacterial microbiota among the vineyards tested.

### Beta diversity of endophytic bacterial microbiota in grapevine shoot xylems

**Figure 5:**
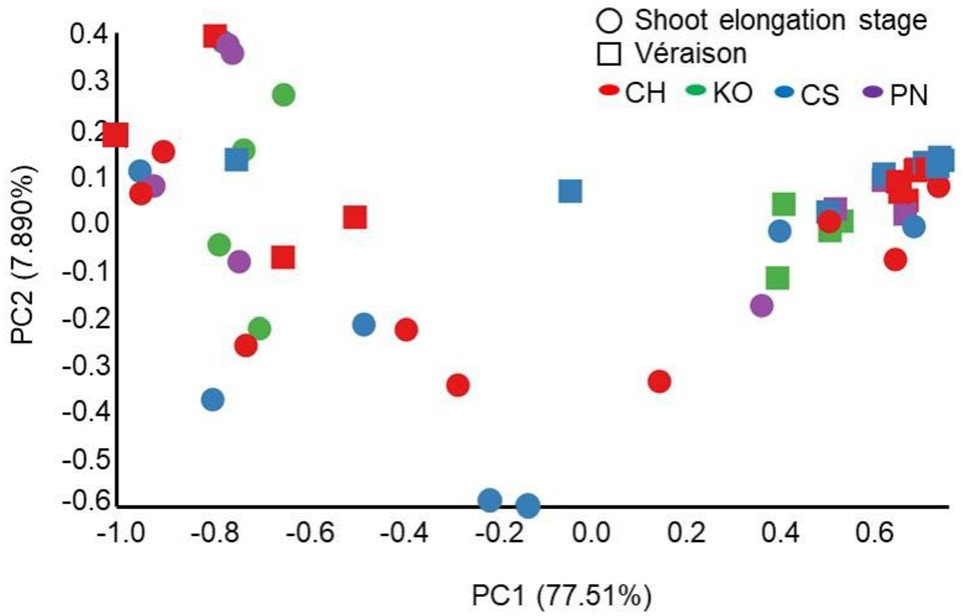
Principal coordinate analysis of endophytic bacterial microbiota in grapevine shoot xylems. Circles (○) and squares (□) indicate endophytic bacterial microbiota at the shoot elongation stage and véraison, respectively. *KO* Koshu, *CH* Chardonnay, *CS* Cabernet Sauvignon, *PN* Pinot Noir.

PCoA demonstrated that the plots of Koshu and Pinot Noir were relatively close to each other at the shoot elongation stage and very close to each other at véraison irrespective of the vineyard (Fig. 5), suggesting that the profiles of endophytic bacterial microbiota in Koshu and Pinot Noir shoot xylems were similar irrespective of both shoot growth stage and vineyard. Although the plots of Chardonnay and Cabernet Sauvignon in each vineyard were widely scattered at the shoot elongation stage, they were very close to each other at véraison. These results suggest that the profiles of endophytic bacterial microbiota in grapevine shoot xylems at véraison were uniform irrespective of the vineyard.

PERMANOVA demonstrated that the *p*-values for all combinations of cultivars exceeded 0.05 (Supplementary Table 4). In contrast, there was a significant difference (*p* = 0.001) between the shoot elongation stage and véraison. Although three of fifty-five combinations of vineyards showed significant differences (*p* = 0.04 for Komoro and Izumo, *p* = 0.007 for Komoro and Kai, and *p* = 0.034 for Kai and Kofu), there was no significant difference between most of the combinations. These results suggest that the variations of endophytic bacterial microbiota in grapevine shoot xylems greatly depended on the shoot growth stage.

### Cluster analysis of endophytic bacterial microbiota in grapevine shoot xylems

**Figure 6:**
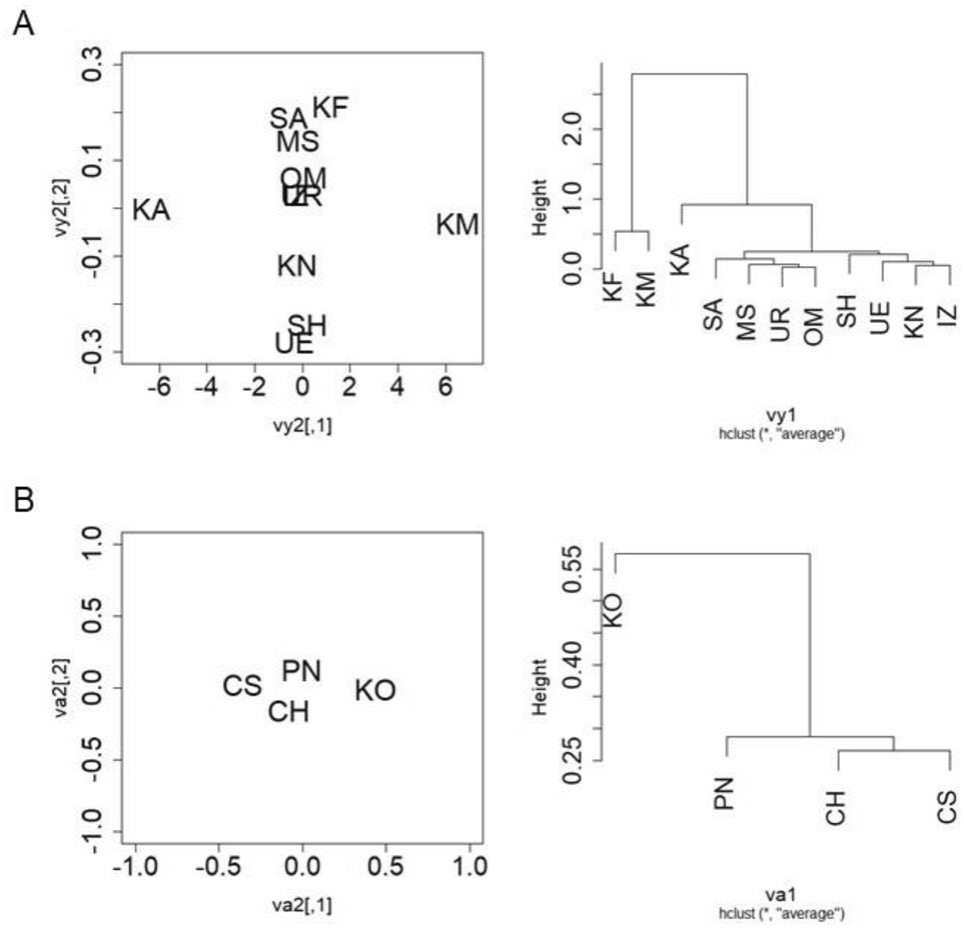
Multidimensional scaling analysis of endophytic bacterial microbiota in grapevine shoot xylems among vineyards or cultivars. (**A**) Vineyards. (**B**) Cultivars. Left, position map. Right, cladogram. *UR* Urausu, *MS* Minamisanriku, *KF* Kofu, *KA* Kai, *KN* Katsunuma, *KM* Komoro, *UE* Ueda, *SH* Shobara, *IZ* Izumo, *SA* Saijo, *OM* Omishima, *KO* Koshu, *CH* Chardonnay, *CS* Cabernet Sauvignon, *PN* Pinot Noir.

**Figure 7:**
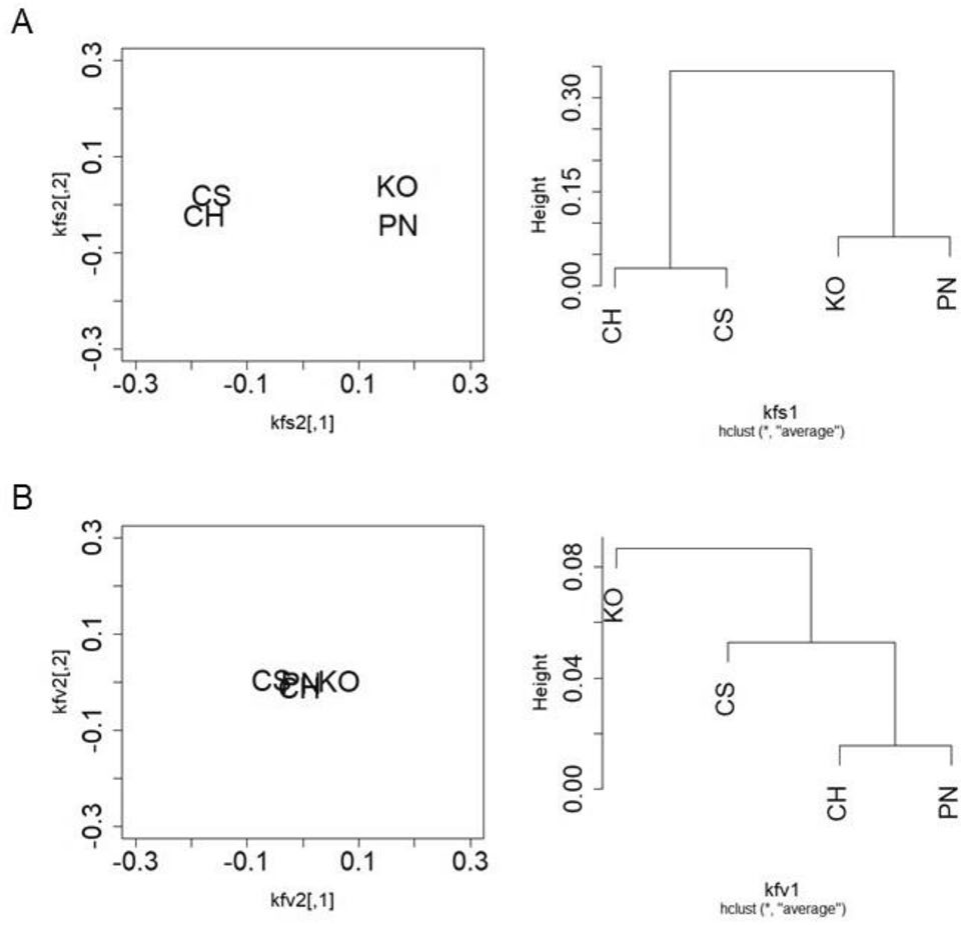
Multidimensional scaling analysis of endophytic bacterial microbiota in grapevine shoot xylems among cultivars grown in Kofu vineyard. (**A**) Shoot elongation stage. (**B**) Véraison. Left, position map. Right, cladogram. *KO* Koshu, *CH* Chardonnay, *CS* Cabernet Sauvignon, *PN* Pinot Noir.

Cluster analysis of endophytic bacterial microbiota in grapevine shoot xylems in various cultivars, shoot growth stages, and vineyards was performed by MDS (Figs. 6 and 7). Cladistic analysis was also conducted using a group average method. Except for Kai and Komoro, nine vineyards were very close to each other in the position map and eight vineyards formed a cluster in the cladogram (Fig. 6A). The four cultivars in the vineyards tested were widely scattered in the position map (Fig. 6B). On the other hand, Koshu and Pinot Noir at the shoot elongation stage, cultivated in Kofu, were close to each other in the position map and formed a cluster in the cladogram (Fig. 7A). Chardonnay and Cabernet Sauvignon at the shoot elongation stage, cultivated in Kofu, were close to each other but apart from Koshu and Pinot Noir, and formed a cluster in the cladogram. Interestingly, at véraison, the four cultivars were very close to each other in the position map (Fig. 7B).

**Figure 8:**
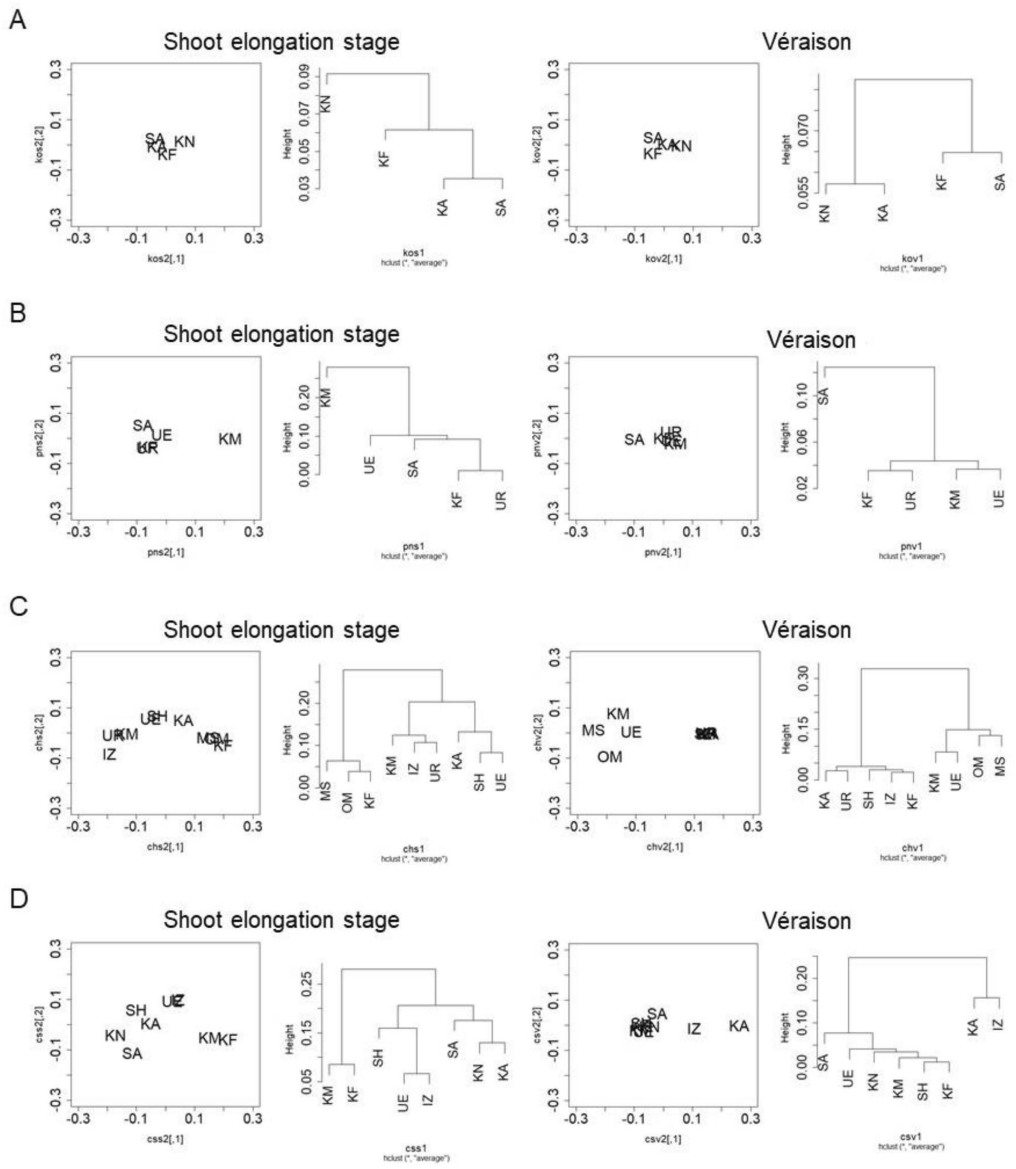
Multidimensional scaling analysis of endophytic bacterial microbiota in grapevine shoot xylems among vineyards cultivating each cultivar. (**A**) Koshu. (**B**) Pinot Noir. (**C**) Chardonnay. (**D**) Cabernet Sauvignon. Left, position map. Right, cladogram. *UR* Urausu, *MS* Minamisanriku, *KF* Kofu, *KA* Kai, *KN* Katsunuma, *KM* Komoro, *UE* Ueda, *SH* Shobara, *IZ* Izumo, *SA* Saijo, *OM* Omishima.

Next, MDS and cladistic analysis of each cultivar in the vineyards were performed (Fig. 8). The distances among vineyards cultivating Koshu were small irrespective of the shoot growth stage (Fig. 8A). The distances among vineyards cultivating Pinot Noir were also small at the shoot elongation stage, and were further decreased at véraison (Fig. 8B). In contrast, the distances among vineyards cultivating Chardonnay and Cabernet Sauvignon were large at the shoot elongation stage (Fig. 8C,D). Although the distances among some vineyards (Urausu, Kofu, Kai, Izumo, and Shobara for Chardonnay, and Ueda, Kofu, Katsunuma, Komoro, and Shobara for Cabernet Sauvignon) decreased at véraison, they were large compared with Koshu and Pinot Noir.

## Discussion

Microbial communities associated with plants play an important role in plant growth, development, and resistance to external stresses^33^. Microbiome analyses in the soil, the rhizosphere, and the phyllosphere of vineyards have been performed at the phylum level^34,35^. In general, Firmicutes, Acidobacteria, Bacteroidetes, and Actinobacteria are predominant in the soil. Proteobacteria is abundant in the rhizosphere, whereas large numbers of Proteobacteria, Firmicutes, Bacteroidetes, and Acidobacteria live on grapevine leaves. In the present study, Proteobacteria (Alphaproteobacteria and Gammaproteobacteria), Firmicutes (Bacilli and Clostridia), Actinobacteria (Actinobacteria), and Bacteroidetes (Bacteroidia) were also identified as endophytic bacteria in the shoot xylems of grapevine. On the other hand, Cyanobacteria (Oxyphotobacteria), which has not been detected in the soil, the rhizosphere, and the phyllosphere of vineyards, existed in the shoot xylems of grapevine. *V. vinifera* cv. Pinot Nero grapevine treated with the extract of Cyanobacteria, *Arthrospira platensis* F&M-C256, isolated from seaweed showed increased berry weight at harvest compared with untreated grapevine^36^. In our previous work, we found that the application of *Bacillus velezensis* KOF112 strain, isolated from the shoot xylems of Koshu, to grapevine leaves induced plant defense response and suppressed downy mildew^37^. Thus, endophytic bacteria in grapevine shoot xylems are promising candidates for biostimulants to confer desirable traits on grapevine. Further exploration of cultivable endophytic bacteria, which prominently affect grape traits, from grapevine shoot xylems is necessary to stabilize grape berry yield and quality, which are affected by environmental conditions.

The morphology of each shoot at the shoot elongation stage differed among vineyards and cultivars. Shoot morphology is mainly affected by the genotype and less by the grapevine-growing region^38^. The mild impact of environmental conditions on shoot morphology suggests stability in the vegetative growth of grapevine. Therefore, the difference in endophytic bacterial microbiota in the shoot xylems tested may be cultivar-dependent and/or soil condition-dependent. We also found that the profiles of endophytic bacterial microbiota in Chardonnay and Cabernet Sauvignon shoot xylems considerably differed among vineyards. A vineyard is a monoculture cropping system, and the lack of plant genotypic diversity across a vineyard may be a bottleneck in soil microbial diversity^21^. In addition, plant roots act as ‘gatekeepers’ to screen soil bacteria from the rhizosphere and the rhizoplane^33^. Regarding Chardonnay and Cabernet Sauvignon, further studies on the relationship between endophytic bacterial microbiota in shoot xylems and soil microbiota in each vineyard are required. On the other hand, the profiles of endophytic bacterial microbiota in the Koshu shoot xylems were highly uniform irrespective of the vineyard, suggesting that endophytic bacterial assemblies in Koshu shoot xylems may not be correlated with environmental conditions in each vineyard including soil microbiota. Koshu is a Japanese indigenous *Vitis* sp. that has been grown for more than a thousand years in Japan^39^. The accumulation of phenylpropanoids and flavonols in Koshu grape skin at harvest is one of the distinct characteristics of Koshu compared with other grape cultivars^40^. The contents of p-coumaric acid, caffeic acid, and flavonols in Koshu grape skin are much higher than those in Chardonnay, Sauvignon Blanc, and Merlot grape skin. The environmental restriction in the grapevine-growing region (in only Japan) for Koshu may have led to the establishment of Koshu-specific endophytic bacterial microbiota in the shoot xylems and contributed to the unique profiles of phenylpropanoids and flavonols in Koshu grape berries. It is necessary to determine the relationship between endophytic bacterial microbiota in Koshu shoot xylems and Koshu grape berry compositions in future experiments.

Analyses of microbiome in grapevine were also performed from the perspective of winemaking. The surface of grape berries has various microorganisms originating from the environment^41^. Commonalities were found between the profiles of microbiota on grape berries and those of soil microbiota in vineyards^42^. Microbiome analyses of grape musts during winemaking demonstrated that the profiles of microbiota in red grape musts differed from those in white grape musts depending on the cultivar, vintage, and climatic conditions^41^. Thus, monochronic microbiome analysis of grapevine tissues may not make sense because of the complexity of microbiota in grapevine related to viticulture and winemaking. Comprehensive microbiome analyses, including endophytic microbiota in grapevine from vineyards to winemaking, would help us understand the impact of microbial biodiversity in grapevine on the characteristics of grape berries and wines.

## Supplementary Information

Supplementary Figures.

Supplementary Tables.

## Data Availability

The sequenced read data were submitted to the DDBJ Read Archive (accession number DRA012630, hold date: August 24, 2023).
