# Supplementary material for: Diversity of endophytic bacterial microbiota in grapevine shoot xylems varies depending on wine grape-growing region, cultivar, and shoot growth stage
Source: Sci Rep. 2022 Sep 21;12:15772. doi: 10.1038/s41598-022-20221-8 (PMC9492663; doi:10.1038/s41598-022-20221-8)
Supplement: Supplementary file 1 — Supplementary Figures. [file 41598_2022_20221_MOESM1_ESM.pptx]

## Slide 1
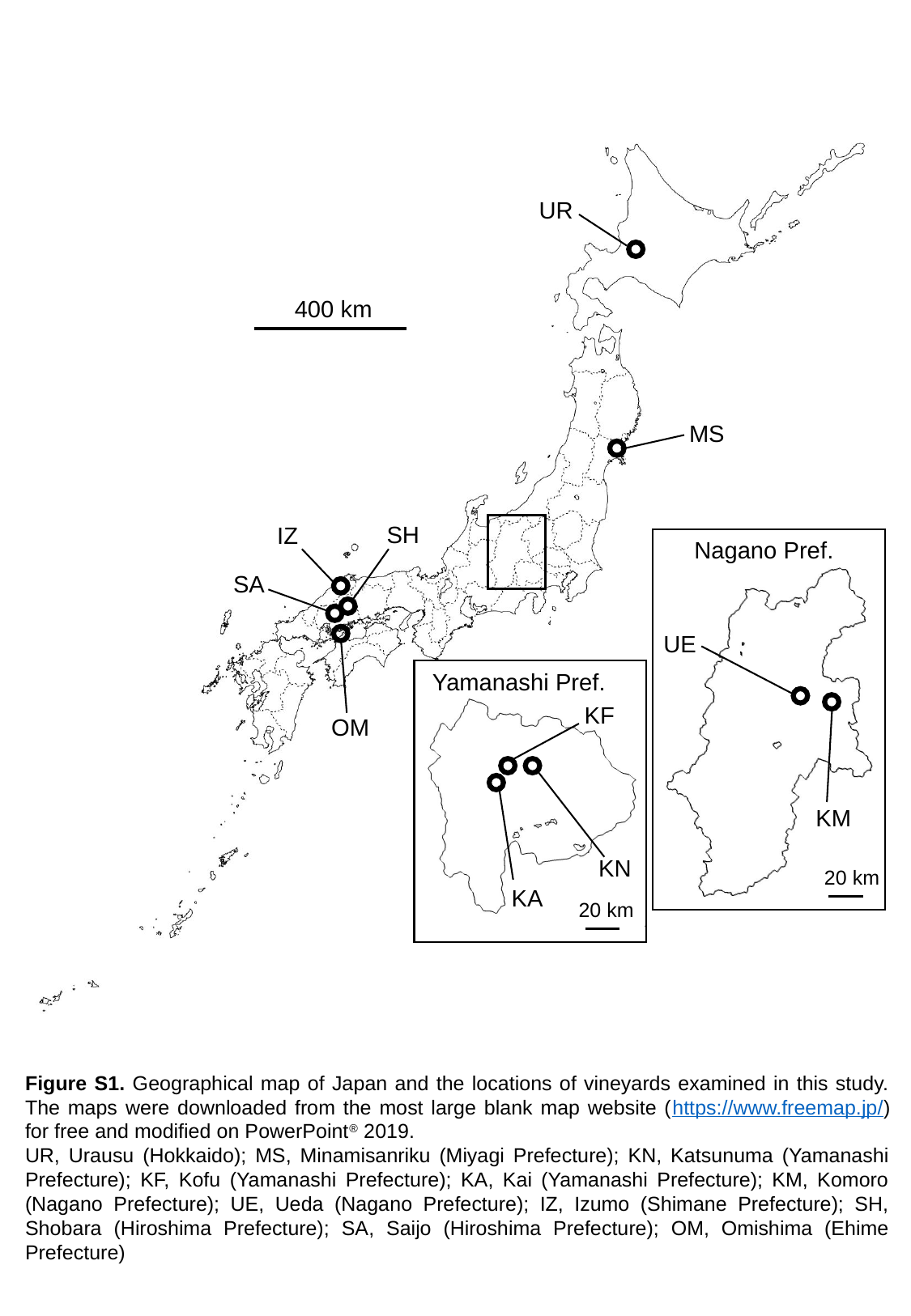

400 km
UR
MS
SH
IZ
Nagano Pref.
SA
UE
Yamanashi Pref.
KF
OM
KM
KN
20 km
KA
20 km
Figure S1. Geographical map of Japan and the locations of vineyards examined in this study. The maps were downloaded from the most large blank map website (https://www.freemap.jp/) for free and modified on PowerPoint® 2019.
UR, Urausu (Hokkaido); MS, Minamisanriku (Miyagi Prefecture); KN, Katsunuma (Yamanashi Prefecture); KF, Kofu (Yamanashi Prefecture); KA, Kai (Yamanashi Prefecture); KM, Komoro (Nagano Prefecture); UE, Ueda (Nagano Prefecture); IZ, Izumo (Shimane Prefecture); SH, Shobara (Hiroshima Prefecture); SA, Saijo (Hiroshima Prefecture); OM, Omishima (Ehime Prefecture)

## Slide 2
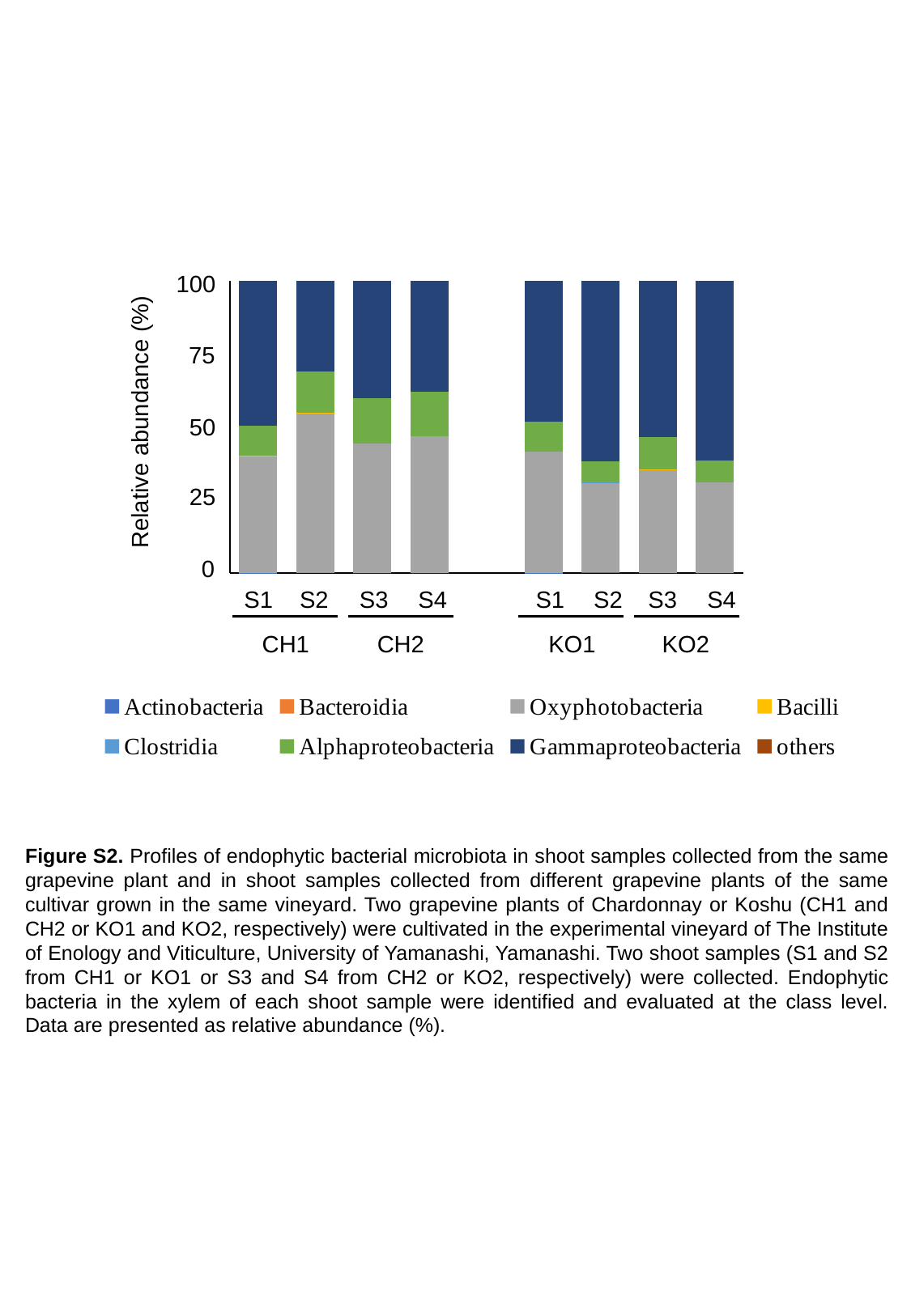

### Chart
| Category | Actinobacteria | Bacteroidia | Oxyphotobacteria | Bacilli | Clostridia | Alphaproteobacteria | Gammaproteobacteria | others |
|---|---|---|---|---|---|---|---|---|
| CH-1-1 | 20.0 | 0.0 | 11600.0 | 7.0 | 0.0 | 2997.0 | 14342.0 | 0.0 |
| CH-1-2 | 76.0 | 11.0 | 18471.0 | 61.0 | 0.0 | 4764.0 | 10503.0 | 0.0 |
| CH-2-1 | 0.0 | 0.0 | 8456.0 | 0.0 | 0.0 | 2970.0 | 7640.0 | 0.0 |
| CH-2-2 | 0.0 | 0.0 | 9667.0 | 0.0 | 0.0 | 3158.0 | 7851.0 | 0.0 |
| | None | None | None | None | None | None | None | None |
| KO-1-1 | 20.0 | 0.0 | 13092.0 | 0.0 | 0.0 | 3193.0 | 15145.0 | 0.0 |
| KO-1-2 | 38.0 | 0.0 | 8505.0 | 0.0 | 31.0 | 2034.0 | 17051.0 | 0.0 |
| KO-2-1 | 0.0 | 0.0 | 6137.0 | 24.0 | 0.0 | 1919.0 | 9290.0 | 0.0 |
| KO-2-2 | 0.0 | 0.0 | 5544.0 | 0.0 | 16.0 | 1262.0 | 10876.0 | 0.0 |100
Relative abundance (%)
75
50
25
0
S1 S2 S3 S4 S1 S2 S3 S4
CH1
CH2
KO1
KO2
### Chart
| Category | Actinobacteria | Bacteroidia | Oxyphotobacteria | Bacilli | Clostridia | Alphaproteobacteria | Gammaproteobacteria | others |
|---|---|---|---|---|---|---|---|---|
| KF | None | None | None | None | None | None | None | None |
| KN | None | None | None | None | None | None | None | None |
| KA | None | None | None | None | None | None | None | None |
| SA | None | None | None | None | None | None | None | None |
| KF | None | None | None | None | None | None | None | None |
| KN | None | None | None | None | None | None | None | None |
| KA | None | None | None | None | None | None | None | None |
| SA | None | None | None | None | None | None | None | None |Figure S2. Profiles of endophytic bacterial microbiota in shoot samples collected from the same grapevine plant and in shoot samples collected from different grapevine plants of the same cultivar grown in the same vineyard. Two grapevine plants of Chardonnay or Koshu (CH1 and CH2 or KO1 and KO2, respectively) were cultivated in the experimental vineyard of The Institute of Enology and Viticulture, University of Yamanashi, Yamanashi. Two shoot samples (S1 and S2 from CH1 or KO1 or S3 and S4 from CH2 or KO2, respectively) were collected. Endophytic bacteria in the xylem of each shoot sample were identified and evaluated at the class level. Data are presented as relative abundance (%).
