# Supplementary material for: Diversity of endophytic bacterial microbiota in grapevine shoot xylems varies depending on wine grape-growing region, cultivar, and shoot growth stage
Source: Sci Rep. 2022 Sep 21;12:15772. doi: 10.1038/s41598-022-20221-8 (PMC9492663; doi:10.1038/s41598-022-20221-8)
Supplement: Supplementary file 2 — Supplementary Tables. [file 41598_2022_20221_MOESM2_ESM.docx]

| Supplementary Table 1 Latitude, longitude, elevation, collection date, and cultivars collected from each vineyard | | | | | | | | | |  |
| --- | --- | --- | --- | --- | --- | --- | --- | --- | --- | --- |
| Vineyard | Latitude | Longitude | Elevation (m) | SES^a^ | V^a^ | KO^b^ | CH^b^ | PN^b^ | CS^b^ | |
| Urausu | 43.45006 | 141.80011 | 90 | June 6 | August 8 |  | ✓ | ✓ |  | |
| Minamisanriku | 38.71311 | 141.40809 | 165 | May 31 | July 27 |  | ✓ |  |  | |
| Katsunuma | 35.6605 | 138.72464 | 375 | May 18 | August 8 | ✓ |  |  | ✓ | |
| Kofu | 35.68005 | 138.5692 | 250 | May 28 | August 24 | ✓ | ✓ | ✓ | ✓ | |
| Kai | 35.71292 | 138.50888 | 535 | May 20 | August 5 | ✓ | ✓ |  | ✓ | |
| Komoro | 36.34272 | 138.41085 | 700 | June 1 | August 26 |  | ✓ | ✓ | ✓ | |
| Ueda | 36.34228 | 138.30103 | 640 | May 25 | August 16 |  | ✓ | ✓ | ✓ | |
| Izumo | 35.28906 | 132.93081 | 160 | May 15 | August 8 |  | ✓ |  | ✓ | |
| Shobara | 34.82639 | 132.97619 | 310 | May 16 | August 17 |  | ✓ |  | ✓ | |
| Saijo | 34.39867 | 132.73544 | 220 | May 20 | August 17 | ✓ |  | ✓ | ✓ | |
| Omishima | 34.24836 | 133.00039 | 5 | May 19 | August 25 |  | ✓ |  |  | |
| ^a^SES, shoot elongation stage; V, véraison | | | | | | | | | |  |
| ^b^KO, Koshu; CH, Chardonnay; PN, Pinot Noir; CS, Cabernet Sauvignon | | | | | | | | | |  |

| Supplementary Table2 GDD, Winkler Index, and precipitation from April 1 to October 31, 2020 in each vineyard | | | |  |
| --- | --- | --- | --- | --- |
| Vineyard | GDD^a^ | Winkler Index | Precipitation (mm) |  |
| Urausu | 1611 | Region II | 1086 |  |
| Minamisanriku | 1721 | Region III | 1330 |  |
| Katsunuma | 2471 | Region V | 1431 |  |
| Kofu | 2471 | Region V | 1431 |  |
| Kai | 2270 | Region V | 1267 |  |
| Komoro | 1947 | Region IV | 989 |  |
| Ueda | 1763 | Region III | 1017 |  |
| Izumo | 2240 | Region V | 1698 |  |
| Shobara | 1947 | Region IV | 1708 |  |
| Saijo | 2112 | Region IV | 1664 |  |
| Omishima | 2240 | Region V | 1520 |  |
| ^a^Growing degree days, base threshold of 10 °C | | | |  |

| Supplementary Table 3 Amplicon sequences collected from each shoot xylem sample | | | | | | | |  |  |
| --- | --- | --- | --- | --- | --- | --- | --- | --- | --- |
| Vineyard | Shoot growth stage | Cultivar | Number of  sequences |  | Vineyard | Shoot growth stage | Cultivar | | Number of  sequences |
| Urausu | SES^a^ | CH^b^ | 46642 |  | Komoro | SES | CH | | 83335 |
| Urausu | SES | PN^b^ | 90677 |  | Komoro | SES | PN | | 77394 |
| Urausu | V^a^ | CH | 197289 |  | Komoro | SES | CS | | 99543 |
| Urausu | V | PN | 201979 |  | Komoro | V | CH | | 151350 |
| Minamisanriku | SES | CH | 70537 |  | Komoro | V | PN | | 141875 |
| Minamisanriku | V | CH | 285003 |  | Komoro | V | CS | | 172319 |
| Katsunuma | SES | KO^b^ | 123836 |  | Ueda | SES | CH | | 76926 |
| Katsunuma | SES | CS^b^ | 110430 |  | Ueda | SES | PN | | 84305 |
| Katsunuma | V | KO | 95462 |  | Ueda | SES | CS | | 83426 |
| Katsunuma | V | CS | 176849 |  | Ueda | V | CH | | 159069 |
| Kofu | SES | KO | 88674 |  | Ueda | V | PN | | 193110 |
| Kofu | SES | CH | 68411 |  | Ueda | V | CS | | 150812 |
| Kofu | SES | PN | 106852 |  | Izumo | SES | CH | | 93828 |
| Kofu | SES | CS | 62628 |  | Izumo | SES | CS | | 87210 |
| Kofu | V | KO | 176566 |  | Izumo | V | CH | | 199041 |
| Kofu | V | CH | 193924 |  | Izumo | V | CS | | 261058 |
| Kofu | V | PN | 228070 |  | Shobara | SES | CH | | 61281 |
| Kofu | V | CS | 226505 |  | Shobara | SES | CS | | 69654 |
| Kai | SES | KO | 102052 |  | Shobara | V | CH | | 206374 |
| Kai | SES | CH | 75394 |  | Shobara | V | CS | | 170910 |
| Kai | SES | CS | 78480 |  | Saijo | SES | KO | | 63692 |
| Kai | V | KO | 155406 |  | Saijo | SES | PN | | 64393 |
| Kai | V | CH | 174877 |  | Saijo | SES | CS | | 70295 |
| Kai | V | CS | 214544 |  | Saijo | V | KO | | 195226 |
|  |  |  |  |  | Saijo | V | PN | | 167782 |
|  |  |  |  |  | Saijo | V | CS | | 209498 |
|  |  |  |  |  | Omishima | SES | CH | | 55749 |
|  |  |  |  |  | Omishima | V | CH | | 219058 |
| ^a^SES, shoot elongation stage; V, véraison | | | | | | | |  |  |
| ^b^CH, Chardonnay; PN, Pinot Noir; KO, Koshu; CS, Cabernet Sauvignon | | | | | | | |  |  |

| Supplementary Table 4 PERMANOVA between vineyards, between cultivars, or between shoot growth stages | | | | | | | | | | |  |
| --- | --- | --- | --- | --- | --- | --- | --- | --- | --- | --- | --- |
| Group 1 | Group 2 | Sample size | pseudo-F | p-value |  | Group 1 | Group 2 | Sample size | pseudo-F | p-value | |
| UR^a^ | IZ^a^ | 8 | 0.130 | 1 |  | KM | UE | 12 | 3.866 | 0.051 | |
| UR | KA^a^ | 10 | 0.969 | 0.364 |  | KM | MS | 8 | 3.012 | 0.147 | |
| UR | KN^a^ | 8 | 0.129 | 0.919 |  | KM | OM | 8 | 2.079 | 0.138 | |
| UR | KM^a^ | 10 | 2.517 | 0.142 |  | KM | SA | 12 | 4.443 | 0.065 | |
| UR | UE^a^ | 10 | 0.222 | 0.869 |  | KM | SH | 10 | 1.599 | 0.210 | |
| UR | MS^a^ | 6 | 0.071 | 0.795 |  | KM | KF | 14 | 0.539 | 0.513 | |
| UR | OM^a^ | 6 | 0.029 | 0.743 |  | UE | MS | 8 | 0.336 | 0.751 | |
| UR | SA^a^ | 10 | 0.132 | 0.701 |  | UE | OM | 8 | 0.216 | 0.800 | |
| UR | SH^a^ | 8 | 0.297 | 0.659 |  | UE | SA | 12 | 0.475 | 0.545 | |
| UR | KF^a^ | 12 | 0.791 | 0.295 |  | UE | SH | 10 | 0.196 | 0.832 | |
| IZ | KA | 10 | 0.805 | 0.459 |  | UE | KF | 14 | 1.486 | 0.241 | |
| IZ | KN | 8 | 0.049 | 1 |  | MS | OM | 4 | 0.060 | 1 | |
| IZ | KM | 10 | 3.418 | 0.040 |  | MS | SA | 8 | 0.188 | 0.574 | |
| IZ | UE | 10 | 0.052 | 0.999 |  | MS | SH | 6 | 0.316 | 0.659 | |
| IZ | MS | 6 | 0.174 | 0.872 |  | MS | KF | 10 | 0.907 | 0.314 | |
| IZ | OM | 6 | 0.184 | 0.938 |  | OM | SA | 8 | 0.127 | 0.759 | |
| IZ | SA | 10 | 0.291 | 0.849 |  | OM | SH | 6 | 0.153 | 0.876 | |
| IZ | SH | 8 | 0.163 | 0.966 |  | OM | KF | 10 | 0.422 | 0.686 | |
| IZ | KF | 12 | 1.080 | 0.309 |  | SA | SH | 10 | 0.553 | 0.506 | |
| KA | KN | 10 | 0.404 | 0.595 |  | SA | KF | 14 | 1.619 | 0.178 | |
| KA | KM | 12 | 12.844 | 0.007 |  | SH | KF | 12 | 0.562 | 0.495 | |
| KA | UE | 12 | 1.318 | 0.212 |  |  |  |  |  |  | |
| KA | MS | 8 | 0.221 | 0.951 |  |  |  |  |  |  | |
| KA | OM | 8 | 0.813 | 0.487 |  | CH^b^ | CS^b^ | 34 | 0.265 | 0.758 | |
| KA | SA | 12 | 1.098 | 0.380 |  | CH | KO^b^ | 26 | 0.512 | 0.545 | |
| KA | SH | 10 | 1.736 | 0.205 |  | CH | PN^b^ | 28 | 0.290 | 0.715 | |
| KA | KF | 14 | 5.239 | 0.034 |  | CS | KO | 24 | 0.793 | 0.412 | |
| KN | KM | 10 | 3.210 | 0.066 |  | CS | PN | 26 | 0.285 | 0.743 | |
| KN | UE | 10 | 0.151 | 0.967 |  | KO | PN | 18 | 0.416 | 0.526 | |
| KN | MS | 6 | 0.105 | 0.853 |  |  |  |  |  |  | |
| KN | OM | 6 | 0.159 | 0.943 |  |  |  |  |  |  | |
| KN | SA | 10 | 0.207 | 0.704 |  | V^c^ | SES^c^ | 52 | 15.844 | 0.001 | |
| KN | SH | 8 | 0.258 | 0.657 |  |  |  |  |  |  |  |
| KN | KF | 12 | 1.202 | 0.239 |  |  |  |  |  |  | |
| ^a^UR, Urausu; MS, Minamisanriku; KN, Katsunuma; KF, Kofu; KA, Kai; KM, Komoro; UE, Ueda; IZ, Izumo;  SH, Shobara; SA, Saijo; OM, Omishima | | | | | | | | | | |  |
| ^b^CH, Chardonnay; PN, Pinot Noir; KO, Koshu; CS, Cabernet Sauvignon | | | | | | | | | | |  |
| ^c^SES, shoot elongation stage; V, véraison | | | | | | | | | | |  |
|  | | | | | | | | | | |  |
